# Fibroblast growth factor 23 levels and the risk of diabetic kidney disease: a systematic review and meta-analysis

**DOI:** 10.3389/fendo.2026.1799186

**Published:** 2026-05-19

**Authors:** Xingjia Wang, Jiameng Li, Xinzhuo Wang, Xiaojian Zhu, Chunyan Liu, Na Zhao, Dan Sun, Jianhui Yuan, Jian Ma

**Affiliations:** 1 Graduate School, Heilongjiang University of Chinese Medicine, Harbin, Heilongjiang, China; 2 The First Clinical Medical College, Heilongjiang University of Chinese Medicine, Harbin, Heilongjiang, China; 3 Department of Endocrinology, The First Affiliated Hospital of Heilongjiang University of Chinese Medicine, Harbin, Heilongjiang, China; 4 Department of Critical Care Medicine, The 2nd Affiliated Hospital of Harbin Medical University, Harbin, Heilongjiang, China

**Keywords:** biomarker, diabetic kidney disease, fibroblast growth factor 23, meta-analysis, renal function, systematic review

## Abstract

**Objective:**

Diabetic kidney disease (DKD) represents one of the most serious microvascular complications associated with diabetes mellitus (DM), and its early diagnosis is crucial for improving prognosis. While fibroblast growth factor 23 (FGF23) has been linked to DKD risk in numerous studies, the existing evidence remains controversial. This study sought to synthesize existing observational data through a systematic review and meta-analysis to clarify the overall association between FGF23 levels and DKD and explore its significance as a potential biomarker.

**Methods:**

This systematic review and meta-analysis followed the Preferred Reporting Items for Systematic Reviews and Meta-Analyses guidelines. A comprehensive search of Cochrane Library, PubMed, Embase, and Web of Science was conducted to identify eligible studies published up to October 23, 2025. We included observational studies that compared FGF23 levels between patients with DKD and non-DKD controls, or evaluated DKD risk. Two researchers independently screened the literature, extracted data, and evaluated methodological quality using the Newcastle–Ottawa Scale (NOS). Statistical analyses were performed with Stata version 15.1. Effect sizes were expressed as standardized mean differences (SMDs) and odds ratios (ORs) with corresponding 95% confidence intervals (CIs). Between-study heterogeneity was quantified using the I² statistic, and subgroup, sensitivity, and publication bias analyses were carried out.

**Results:**

Nine eligible studies involving 3,799 participants were included. Meta-analysis indicated that serum FGF23 levels were markedly elevated in patients with DKD compared with those without DKD (SMD = 1.144, 95% CI: 0.361 to 1.928, p = 0.004). Although the pooled OR for DKD with high FGF23 levels was 1.136 (95% CI: 0.656 to 1.968), this association was not statistically significant in dichotomous variable analysis (p = 0.649), with substantial heterogeneity across studies (I² > 80%). Subgroup analyses indicated that region, control type, publication year, DM type, and DKD stage did not fully explain the observed heterogeneity. Sensitivity analyses confirmed the robustness of the mean difference results.

**Conclusion:**

FGF23 levels are associated with the progression of DKD, suggesting that it may reflect metabolic disturbances associated with DKD. Given that the OR analysis did not show statistical significance and heterogeneity was substantial, this association should be considered exploratory.

**Systematic Review Registration:**

https://www.crd.york.ac.uk/prospero/, identifier CRD420251180089.

## 1 Introduction

As one of the most serious microvascular complications of diabetes mellitus (DM), diabetic kidney disease (DKD) represents the predominant contributor to end-stage renal disease (ESRD) globally now. It is also widely recognized as a principal driver of chronic kidney disease (CKD) (1–3), significantly increasing the risk of cardiovascular disease and mortality (4–10). Timely diagnosis and management of DKD represent crucial steps if patient prognosis is to be improved. However, traditional biomarkers such as proteinuria and estimated glomerular filtration rate (eGFR) exhibit limited sensitivity and specificity for detecting early-stage disease (5). In recent years, increasing attention has been directed toward fibroblast growth factor 23 (FGF23), a bone-derived endocrine factor involved in mineral homeostasis, which has been found to be markedly elevated in CKD and closely associated with renal function decline (4, 11–15). Secreted mainly by osteocytes, FGF23 modulates calcium–phosphate balance by inhibiting renal tubular phosphate reabsorption and vitamin D activation (11, 16). In the diabetic milieu, hyperglycemia and insulin resistance may further disrupt FGF23 secretion and metabolism, suggesting a potential mechanistic link between FGF23 and DKD pathogenesis (11, 17).

Despite accumulating evidence suggesting an association between FGF23 and DKD risk, its precise role and clinical significance remain controversial. Whether the relationship between FGF23 and DKD is independent of traditional risk factors has not been fully established. Isakova et al. proposed that elevated FGF23 levels are primarily driven by reduced eGFR and do not support FGF23 functioning as an independent predictor in patients with type 2 diabetes mellitus (T2DM) complicated by CKD (18). Lee et al. similarly suggested that the predictive value of FGF23 for DKD is not independent but may be mediated or replaced by tumor necrosis factor receptor 1 (TNFR1) (19). In contrast, a prospective study by Liu et al. demonstrated that FGF23 is an independent predictor of DKD in patients with T2DM and increases progressively with disease progression (4). Kang et al. also reported that FGF23 could serve as a predictive biomarker for DKD (20). Moreover, discrepancies persist regarding the risk of DKD associated with elevated FGF23 levels. A decreased likelihood of DKD in individuals with higher FGF23 levels was reported by Farías-Basulto et al. (21), whereas Zou et al. (5) observed that elevated FGF23 levels significantly increased the risk of DKD diagnosis, further underscoring the complexity of FGF23’s role in DKD.

Based on these controversies, the present study aimed to integrate existing evidence through a systematic review and meta-analysis in order to better define the relationship between FGF23 levels and the risk of DKD and to explore heterogeneity across different diabetes types, disease stages, and geographic regions. The findings may provide further exploratory evidence for the potential role of FGF23 as a potential biomarker for DKD and help to enhance understanding of its potential mechanisms in DKD progression. In addition, this study may provide preliminary insights into potential therapeutic targets within the FGF23 signaling pathway that could potentially contribute to improved renal outcomes, although this remains speculative (5, 17, 20, 22, 23).

## 2 Methods

This meta-analysis was performed in line with the Preferred Reporting Items for Systematic Reviews and Meta-Analyses (PRISMA) guidelines (24), and was prospectively registered in the International Prospective Register of Systematic Reviews (PROSPERO) (CRD420251180089).

### 2.1 Literature search

A systematic literature search was performed in PubMed, Cochrane Library, Embase, and Web of Science from their inception to October 23, 2025, with no restrictions on language. Search terms included “diabetic nephropathy,” “diabetic glomerulosclerosis,” “diabetic kidney disease,” “diabetic nephropath,” “diabetic renal disease,” “intracapillary glomerulosclerosis,” “Kimmelstiel Wilson Disease,” “Kimmelstiel Wilson Syndrome,” “Nodular Glomerulosclerosis,” “FGF 23,” “FGF23,” “fibroblast growth factor 23,” “phosphatonin,” “tumor derived hypophosphatemia factor,” “tumor derived hypophosphatemia inducing factor,” “tumour derived hypophosphataemia factor,” and “tumour derived hypophosphataemia inducing factor.” Both Medical Subject Headings (MeSH) and free-text keywords were combined using Boolean operators (AND, OR). Full details of the search strategy are provided in File S1</xr>. In addition, reference lists of all eligible articles were also manually screened.

### 2.2 Inclusion and exclusion criteria

Studies were included if they met all of the following criteria: a. Participants were patients with DKD; b. The exposure of interest was FGF23 levels; c. The control group consisted of non-DKD individuals; d. Outcomes included FGF23 levels or risk estimates for DKD; e. The study design was observational.

Studies were excluded if they were meta-analyses, reviews, conference abstracts, animal studies, guidelines, letters, commentaries, inappropriate exposure definitions, case reports, irrelevant study objectives or diseases, inability to access full texts, or insufficient data for extraction.

### 2.3 Literature screening

Two researchers (Xingjia Wang and Jian Ma) independently conducted the preliminary screening of each record in the database using Endnote X9 software according to the predefined inclusion and exclusion criteria. They reviewed the titles and abstracts, and for records that appeared to meet the criteria, full-text articles were retrieved for further assessment. During the full-text review stage, the researchers read the complete articles to make a final judgment on eligibility. Data extraction was performed for articles meeting the criteria. In cases of disagreement between the two primary researchers, a consensus was reached through discussion with a third researcher (Jiameng Li).

### 2.4 Data extraction

Preparation for data collection involved the development of a standardized data extraction form. Two investigators (Xingjia Wang and Xiaojian Zhu) independently collected information from each eligible study, including: author, publication year, study design, country, sample size, control type, age, sex distribution, DKD stage, diabetes type, diabetes duration, body mass index (BMI), glycated hemoglobin (HbA1c), eGFR, serum phosphate (PO4), uric acid, urinary albumin-to-creatinine ratio (UACR), follow-up duration, and outcome measures (means with standard deviations, ORs with 95% CIs). When disagreements arose, they were referred to a third investigator (Xinzhuo Wang) for final determination.

### 2.5 Quality assessment

Study quality was independently assessed by two reviewers (Xingjia Wang and Jianhui Yuan) who applied the Newcastle–Ottawa Scale (NOS) (25). The NOS assesses studies based on selection, comparability, and exposure, with maximum scores of 4 stars, 3 stars, and 2 stars, respectively, totaling 9 stars. The following quality classification was applied: high quality (7–9 stars), moderate quality (4–6 stars), and low quality (≤3 stars). A third investigator (Jian Ma) was consulted to resolve any discrepancies.

### 2.6 Statistical analysis

Stata version 15.1 software was employed to carry out statistical analyses. Pooled SMDs and ORs with corresponding 95% CIs were calculated. Heterogeneity was assessed using the I² statistic; an I² > 50% and p < 0.1 indicated substantial heterogeneity, warranting the use of a random-effects model; otherwise, a fixed-effects model was applied. Subgroup analyses were performed by region, control type, DKD stage, publication year, and diabetes type. Sensitivity analysis was also conducted by sequentially excluding each study to observe whether the results remained within the original 95% confidence interval, thereby verifying result stability. We employed funnel plots along with Egger’s test to assess publication bias, with statistical significance defined as a p-value less than 0.05.

## 3 Results

### 3.1 Results of literature screening

**Figure 1:**
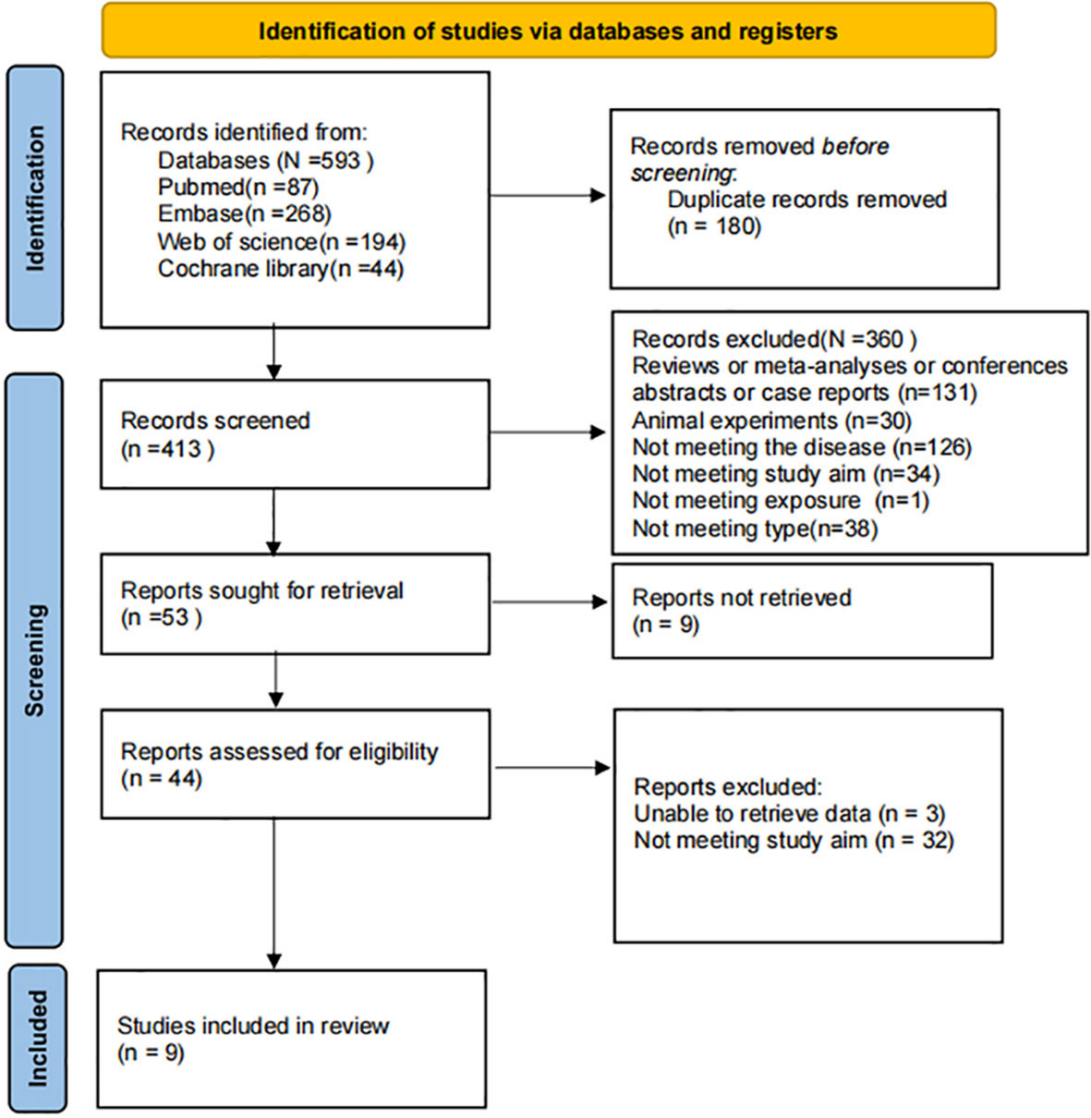
Literature screening flowchart.

Of the 593 articles identified from the search, 180 duplicates were removed, 360 were excluded during initial screening by reviewing titles and abstracts. Ultimately, a full-text review of the remaining articles excluded a further 44, resulting in the final inclusion of nine articles. A flowchart detailing this process is presented in **Figure 1**.

### 3.2 Characteristics of the included studies

A total of nine case–control studies, published from 2013 to 2025, were incorporated in the final analysis (4, 5, 18–21, 26–28). Among them, 1 study was from Mexico, 1 from Egypt, 2 from Turkey, 2 from the United States, and 3 from China, involving a total of 3799 patients. The mean age ranged from 40.30 to 62.39 years, with males accounting for 46.88% of participants. DKD stages ranged from early disease to end-stage renal disease. The mean duration of diabetes ranged from 2.96 to 12.97 years. Detailed baseline characteristics are presented in **Table 1**.

**Table 1:** Baseline information of the included studies.

Author	Year	Design	Country	Sample size	DKD cases	Control type	Age	Sex (M/F)	DKD stage	Diabetes type	Diabetes duration (years)	BMI	HbA1c	eGFR	Serum phosphate (PO4)	Uric acid	UACR	25-Hydroxyvitamin D	PTH	Detection methods for FGF23	Fasting state	Storage condition	Follow-up	Outcomes
Farías-Basulto	2018	Case-control	Mexico (America)	136	52	Diabetic without DKD	60.8012(± 3.6552)	48/88	Early	NA	9.3169(± 2.8784)	29.6176(± 1.1484)(kg/m^2^)	7.5645(± 0.8711)%	98.5175(± 5.3151)(mL/min)	NA	5(± 0.4215) (mg/dL)	47.5511(± 55.0889) (mg/g)	NA	NA	NA	Yes	-80°C	NA	Mean, OR
Ozdemir	2023	Case–control	Turkey (Asia)	76	38	Healthy controls	49.75(± 13.7175)	39/37	NA	NA	11.4(± 9.7)	29.6049(± 10.1576)(kg/m^2^)	7.3522(± 2.7681)%	85.5081(± 38.84) (mL/min)	4.1918(± 1.4882) (mg/dL)	5.455(± 1.5366)(mg/dL)	NA	11.072(± 8.1503)(ng/mL)	74.6088(± 55.3374)(pg/mL)	NA	Yes	-80°C	NA	Mean
Isakova	2015	Case–control	America and Canada (America)	1110	644	Diabetic without DKD	62.3863(± 6.3695)	555/555	NA	NA	9.4206(± 1.5465)	32.6802(± 5.5474)(kg/m^2^)	8.216(± 1.0044)%	86.4477(± 19.4707)(mL/min)	3.458(± 0.5022)(mg/dL)	NA	8.7991(± 1.5863)(mg/g)	NA	NA	Intact	NA	NA	4.7	Mean
Liu	2025	Case–control	China (Asia)	1415	198	Diabetic without DKD	62.1907(± 3.954)	551/864	NA	NA	2.9601(± 0.9241)	25.934(± 3.3442)(kg/m^2^)	6.7132(± 1.1775)%	96.5269(± 2.2091)(mL/min)	1.1028(± 0.1587)(mmol/L)	NA	7.6978(± 2.1051)(mg/g)	NA	NA	Intact	Yes	-80°C	4.6	Mean, OR
Zou	2025	Case–control	China(Asia)	348	121	Diabetic without DKD	54.3612(± 3.0498)	209/139	NA	Type 1	NA	24.5071(± 0.8756)(kg/m^2^)	9.0176(± 0.6284)%	113.9764(± 4.6357)(mL/min)	NA	292.6858(± 22.4919)(umol/L)	1.8665(± 0.6988)(mg/g)	NA	NA	NA	Yes	-80°C	2	Mean, OR
Kang	2024	Case–control	China(Asia)	126	96	Diabetic without DKD	59.5002(± 11.2946)	80/46	Stages III–V	Type 2	NA	25.5541(± 1.407)(kg/m^2^)	NA	60.4539(± 38.4254)(mL/min)	1.3419(± 0.3996)(mmol/L)	373.8469(± 61.6958)(umol/L)	NA	31.2866(± 17.3296)(nmol/L)	102.3679(± 101.0262)(pg/mL)	Intact	Yes	-80°C	NA	Mean
Lee	2013	Case–control	America (America)	297	48	Diabetic without DKD	54.9697(± 9.8213)	157/140	Stage V	Type 2	12.9697(± 8.0145)	NA	8.397(± 1.6815)%	93.5354(± 30.7256)(mL/min)	NA	NA	NA	NA	NA	C-terminal	NA	-80°C	8-12	Mean, HR
Inci	2016	Case–control	Turkey (Asia)	141	109	Healthy controls	58.8839(± 10.5532)	74/67	Stages I–IV	Type 2	NA	30.1221(± 4.9496)(kg/m^2^)	NA	65.0723(± 26.3233)(mL/dak)	NA	NA	202.1962(± 308.961)(mg/d)	27.0335(± 23.9232) (ng/mL)	87.5833(± 99.8319) (pg/mL)	NA	Yes	-80°C	NA	Mean
Fayed	2023	Case–control	Egypt (Africa)	150	50	Healthy + diabetic without DKD	40.3041(± 13.0862)	68/82	NA	NA	NA	23.8128(± 1.4849)(kg/m^2^)	5.9708(± 0.8527)%	87.4913(± 43.6273)(mL/min)	4.0292(± 0.1344)(mg/dL)	5.6374(± 1.0525)(mg/dL)	44.7369(± 51.0432)(mg/g)	25.2456(± 8.4581)(ng/mL)	61.7836(± 10.9731)(ng/mL)	Intact	Yes	NA	NA	Mean

### 3.3 Quality assessment

Based on the NOS assessment, 8 studies were of high quality, and 1 study (21) was of moderate quality. This was attributed to slightly lower scores in case representativeness, control selection, and comparability. Specifically, it was not clearly stated whether consecutive cases were included, community controls were used, and comparability was not adequately addressed by controlling for confounding factors. The specific assessment results are shown in **Table 2**.

**Table 2:** Quality assessment of the included studies.

Study	Selection				Comparability	Exposure			Overall score
	Is the case definition adequate?	Representativeness of the cases	Selection of controls	Definition of controls	Comparability of cohorts based on the design or analysis	Ascertainment of exposure	Same method of ascertainment for cases and controls	Non-response rate	
Farías-Basulto2018	**☆**	**-**	**-**	**☆**	**☆**	**☆**	**☆**	**☆**	6
Ozdemir2023	**☆**	**☆**	**-**	**☆**	**☆**	**☆**	**☆**	**☆**	7
Isakova2015	**☆**	**☆**	**-**	**☆**	**☆**	**☆**	**☆**	**☆**	7
Liu2025	**☆**	**☆**	**☆**	**☆**	**☆ ☆**	**☆**	**☆**	**☆**	9
Zou2025	**☆**	**☆**	**-**	**☆**	**☆**	**☆**	**☆**	**☆**	7
Kang2024	**☆**	**☆**	**-**	**☆**	**☆ ☆**	**☆**	**☆**	**☆**	8
Lee2013	**☆**	**☆**	**-**	**☆**	**☆ ☆**	**☆**	**☆**	**☆**	8
Inci2016	**☆**	**☆**	**-**	**☆**	**☆**	**☆**	**☆**	**☆**	7
Fayed2023	**☆**	**☆**	**-**	**☆**	**☆**	**☆**	**☆**	**☆**	7

The symbol (**☆**) are used to assign scores for assessing the quality of the included studies. The total number of stars for each included study is summed to obtain a total score, which is used to judge the quality of the included studies.

### 3.4 Meta-analysis

#### 3.4.1 Mean difference analysis

**Figure 2:**
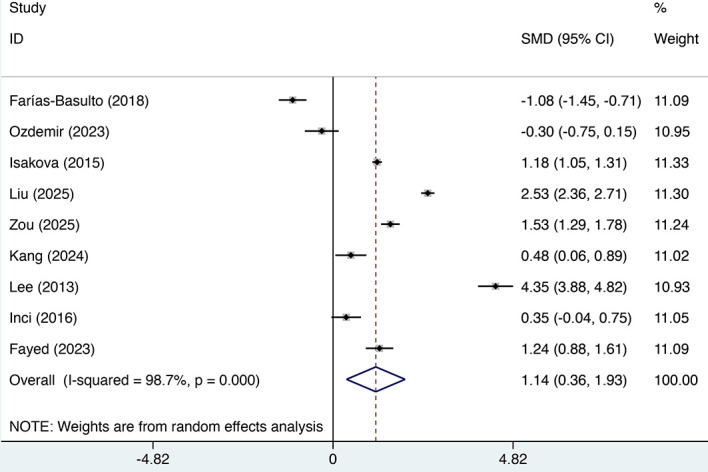
Forest plot of standardized mean differences in FGF23 levels between DKD and non-DKD groups.

Nine studies reported differences in mean FGF23 levels between DKD and non-DKD groups. Substantial heterogeneity was observed (I² = 98.7%, p < 0.01), and subgroup analyses failed to explain this heterogeneity. Therefore, a random-effects model was employed. The pooled estimates, as shown in **Figure 2**, revealed a significant elevation in serum FGF23 levels among DKD patients compared to non-DKD controls (SMD = 1.144, 95% CI: 0.361 to 1.928, p = 0.004). However, given the substantial and largely unexplained heterogeneity across studies, the potential role of FGF23 should be considered exploratory.

Subgroup analyses were performed to explore heterogeneity, stratifying data by region, control type, publication year, diabetes type, and DKD stage (**Table 3**). After stratification by region, control type, year, diabetes type, and DKD stage, subgroup results generally indicated a trend toward higher FGF23 levels in the DKD group than in the non-DKD group across all subgroups: American population (SMD = 1.48, 95% CI: -0.79 to 3.74), Asian population (SMD = 0.93, 95% CI: -0.12 to 1.98), African population (SMD = 1.24, 95% CI: 0.88 to 1.61), healthy controls (SMD = 5.12, 95% CI: 1.73 to 8.51), diabetes-only controls (SMD = 1.31, 95% CI: 0.40 to 2.23), studies before 2020 (SMD = 1.19, 95% CI: -0.46 to 2.84), studies after 2020 (SMD = 1.11, 95% CI: 0.16 to 2.06), type 1 diabetes patients (SMD = 1.53, 95% CI: 1.29 to 1.78), type 2 diabetes patients (SMD = 1.72, 95% CI: -0.72 to 4.17), DKD stage 1 (SMD = 0.3, 95% CI: -0.48 to 1.08), DKD stage 2 (SMD = 0.66, 95% CI: 0.14 to 1.18), DKD stage 3 (SMD = 0.15, 95% CI: -0.18 to 0.48), DKD stage 4 (SMD = 0.56, 95% CI: 0.18 to 0.95), and DKD stage 5 (SMD = 2.61, 95% CI: -0.80 to 6.02). However, these factors did not account for the source of heterogeneity. It should be noted that the mean difference analysis in this study was not adjusted for renal function (e.g., eGFR), and thus the potential confounding effect of this factor was not controlled. Besides, the heterogeneity observed in this study may be attributable to multiple factors, including differences in assay methods, sampling timing, and storage conditions. Given the substantial inconsistencies across studies, direct comparisons are challenging, and the findings presented herein should be regarded as preliminary estimates of overall trends. Therefore, the results should be interpreted with caution.

**Table 3:** Subgroup analysis for mean difference in FGF23 between DKD and non-DKD groups.

SMD	Number of studies	SMD	95%CI	p	I^2^,p
Region	9	1.14	(0.36, 1.93)	0.004	98.7%,p<0.001
Americas	3	1.48	(-0.79, 3.74)	0.202	99.4%,p<0.001
Asia	5	0.93	(-0.12, 1.98)	0.081	98.3%,p<0.001
Africa	1	1.24	(0.88, 1.61)		
Control Type	10	1.97	(1.10, 2.85)	<0.001	98.9%,p<0.001
Healthy controls	7	5.12	(1.73, 8.51)	0.003	99.0%,p<0.001
Diabetic without DKD controls	3	1.31	(0.40, 2.23)	0.005	98.9%,p<0.001
DKD Stage	8	0.96	(-0.11, 2.02)	0.079	97.0%,p<0.001
DKD1	1	0.3	(-0.48, 1.08)	0.45	
DKD2	1	0.66	(0.14, 1.18)	0.013	
DKD3	2	0.15	(-0.18, 0.48)	0.363	0.0%,p=0.741
DKD4	2	0.56	(0.18, 0.95)	0.004	0.0%,p=0.822
DKD5	2	2.61	(-0.80, 6.02)	0.133	98.9%,p<0.001
Publication Year	9	1.14	(0.36, 1.93)	0.004	98.7%,p<0.001
Before 2020	4	1.19	(-0.46, 2.84)	0.156	99.1%,p<0.001
2020 and later	5	1.11	(0.16, 2.06)	0.021	98.0%,p<0.001
Diabetes Type	4	1.67	(0.18, 3.17)	0.029	98.5%,p<0.001
T1DM	1	1.53	(1.29, 1.78)	<0.001	
T2DM	3	1.72	(-0.72, 4.17)	0.167	99.0%,p<0.001

#### 3.4.2 Risk ratio analysis

**Figure 3:**
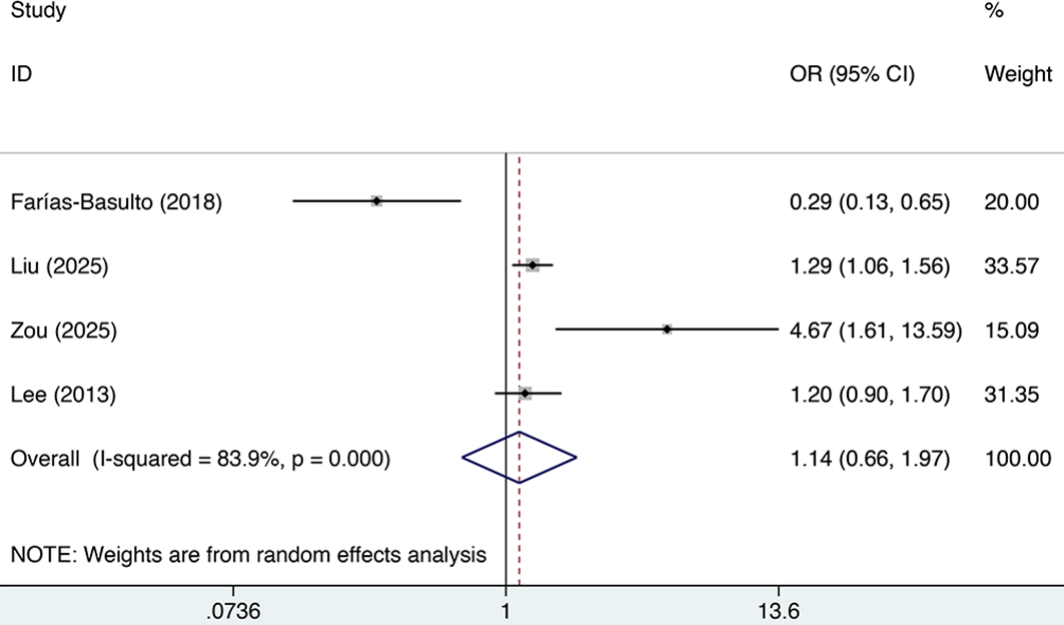
Forest plot of OR for the risk of DKD with high FGF23 levels.

The association between elevated FGF23 levels and DKD risk was reported in four included studies. Due to the presence of significant heterogeneity (I² = 83.9%, p < 0.01), a random-effects model was used. **Figure 3** shows the pooled result: a non-significant OR of 1.136 (95% CI: 0.656 to 1.968, p = 0.649). Given the limited number of included studies, the interpretation of these negative findings should be approached with caution. Given the substantial and largely unexplained heterogeneity across studies, the potential role of FGF23 should be considered exploratory.

In order to explore potential sources of heterogeneity, subgroup analyses were performed, detailed in **Table 4**. We stratified by region and year. However, high FGF23 levels were not associated with DKD in these subgroups, indicating that neither region nor year were sources of heterogeneity. However, the heterogeneity identified in this study may arise from various factors, including differences in assay methods, sampling timing, and storage conditions. Given the marked variability among studies, direct comparisons are constrained, and the present findings should be considered as preliminary estimates of overall trends. Accordingly, cautious interpretation of the results is warranted.

**Table 4:** Subgroup analysis for OR of DKD risk with high FGF23 levels.

Subgroup	The number of studies	OR	95%CI	p	I^2^,p
Region	4	1.14	(0.66, 1.97)	0.460	83.9%,p<0.001
Americas	2	0.62	(0.15, 2.49)	0.500	90.3%,p=0.001
Asia	2	2.2	(0.63, 7.61)	0.215	81.5%,p=0.020
Publication Year	4	1.14	(0.66, 1.97)	0.649	83.9%,p<0.001
Before 2020	2	0.62	(0.15, 2.49)	0.5	90.3%,p=0.001
2020 and later	2	2.2	(0.63, 7.61)	0.21	81.5%,p=0.020

### 3.5 Sensitivity analysis and publication bias

**Figure 4:**
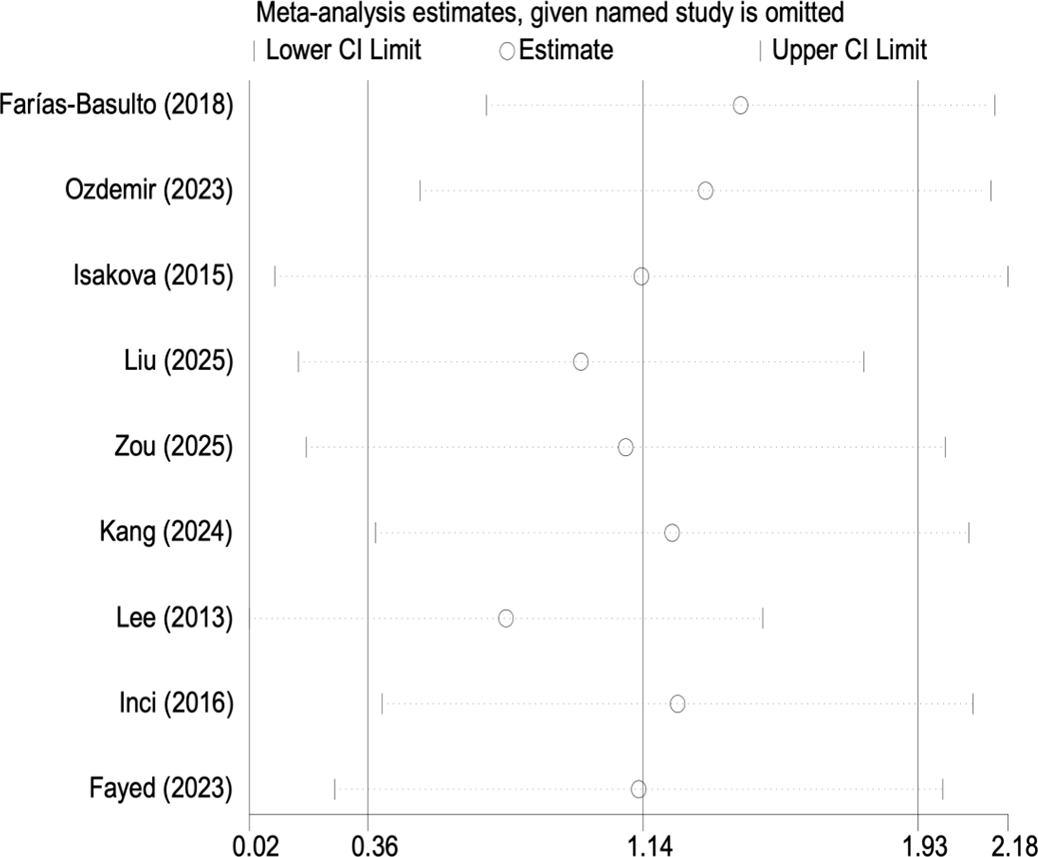
Sensitivity analysis for mean difference in FGF23 between DKD and non-DKD groups.

**Figure 5:**
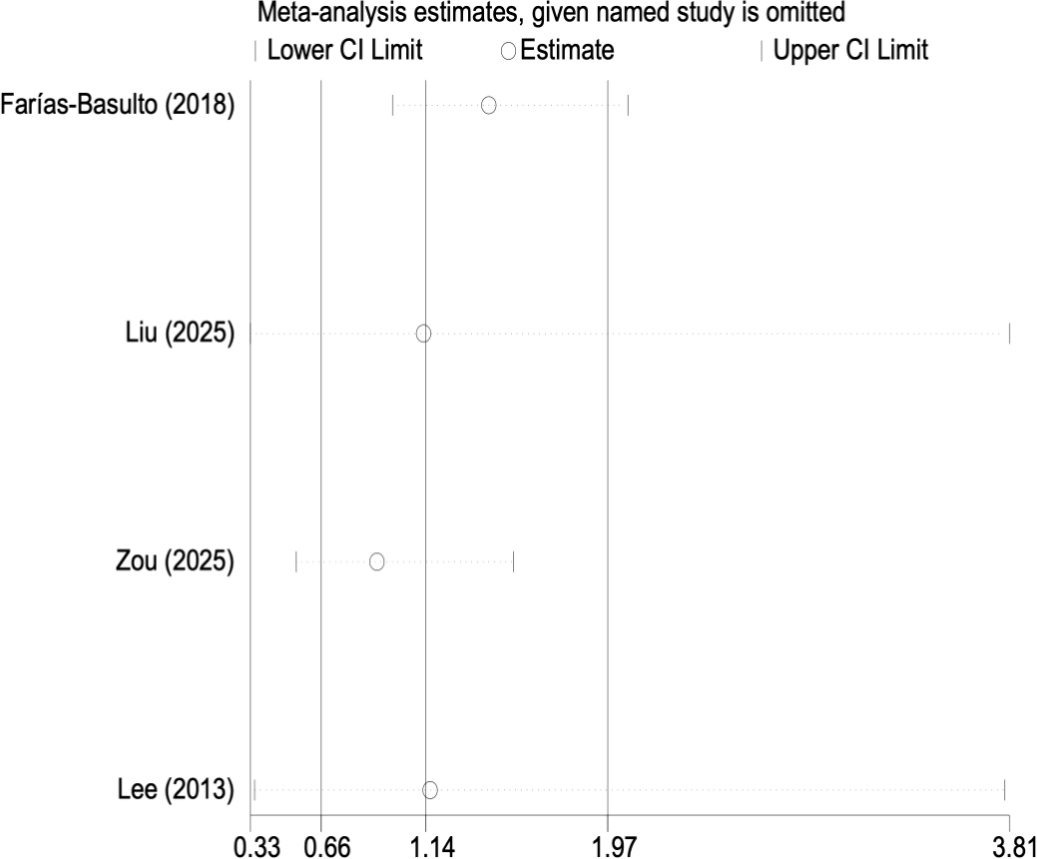
Sensitivity analysis for OR of FGF23 between DKD and non-DKD groups.

The robustness of the pooled effect size was confirmed by sensitivity analysis, in which the sequential exclusion of individual studies did not alter its direction or statistical significance. These results are presented in **Figures 4**, **5**.

**Figure 6:**
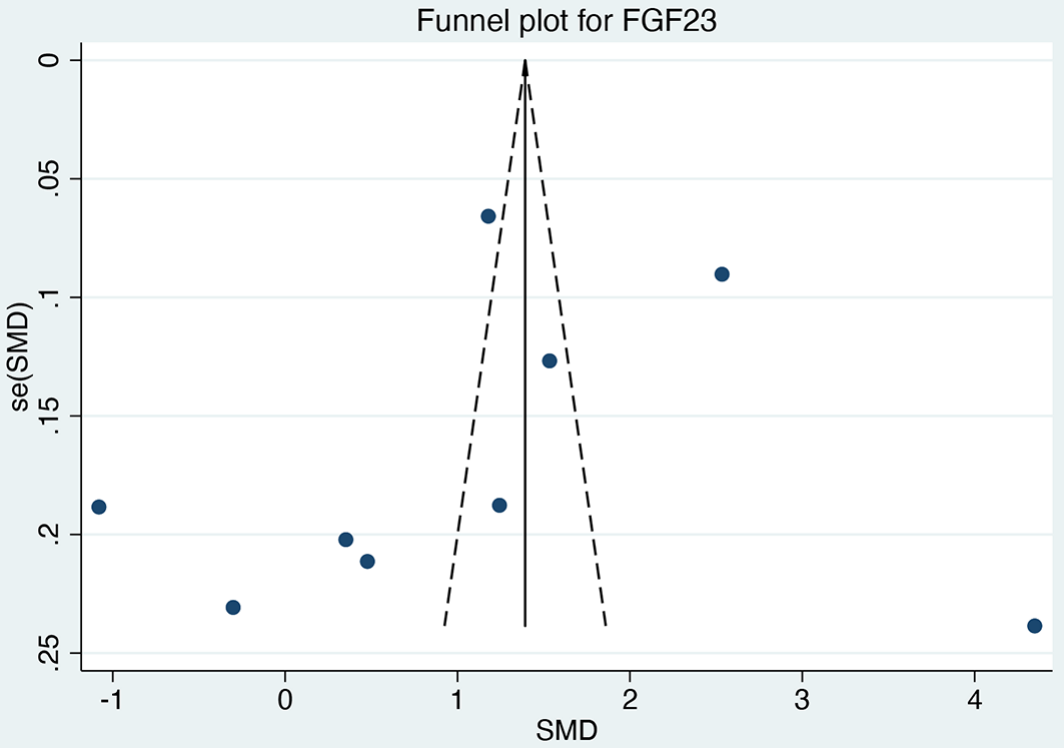
Funnel plot for mean difference in FGF23 between DKD and non-DKD groups.

Visual inspection of the funnel plot (**Figure 6**) suggested approximate symmetry, a finding supported by a non-significant result on Egger’s test (p = 0.544). This indicates a low probability of substantial publication bias for the mean difference analysis. However, due to the limited number of studies reporting odds ratios (ORs), publication bias assessment was not performed for the OR analysis.

## 4 Discussion

Employing a dual approach that examines both “level differences” and “risk prediction,” this systematic review and meta-analysis is the first to comprehensively delineate the complex link between FGF23 and DKD. Synthesizing data from nine observational studies worldwide (involving 3,799 participants up to October 2025), it stands as one of the most extensive quantitative assessments to date. The findings confirm that FGF23 levels are associated with the progression of DKD. However, the extremely high and largely unexplained heterogeneity observed in the continuous variable analysis suggests that the pooled effect estimates may vary across different study designs and populations. Therefore, all findings—particularly those related to biomarker associations and potential clinical implications—should be interpreted with caution and considered exploratory rather than confirmatory. This represents a notable limitation of the present study.

An intriguing finding emerged in this study: while continuous variable analysis showed significantly higher FGF23 levels in DKD patients, dichotomized analyses (high vs. low FGF23) did not yield statistically significant ORs. This apparent discrepancy reflects the combined effects of statistical methodology, biological characteristics, and study design. Continuous variable analyses preserve the full distributional information of FGF23 and are more sensitive to overall shifts between groups, whereas artificially categorizing continuous variables inevitably leads to information loss and reduced statistical power, especially when sample sizes are limited or data distribution is highly skewed. This processing can easily dilute the effect, as has been well-documented in previous studies of biomarkers for chronic kidney disease (29, 30). From a biological perspective, FGF23 is more likely a continuously increasing stress hormone in response to progressive renal dysfunction rather than a binary “on–off” risk factor that triggers DKD once a threshold is crossed. FGF23 levels have been shown to be inversely associated with eGFR in prior studies, with elevations reflecting compensatory responses to phosphate retention, inflammation, and disordered mineral metabolism (12, 31). From a physiological perspective, FGF23 is inherently a continuous variable rather than a simple dichotomous one. Therefore, its continuous nature should be respected in analytical frameworks. For instance, evaluating the risk change associated with each unit (or standard deviation) increase in FGF23 provides greater biological interpretability and statistical power than arbitrarily categorizing it into high and low levels. Such a dose–response relationship more accurately reflects the link between elevated FGF23 concentrations and the pathophysiological progression of DKD. Accordingly, future studies are recommended to prioritize continuous data analysis to more precisely assess this relationship. Therefore, FGF23’s role in DKD progression aligns more with a “dose-response” model than a binary “presence or absence” model, explaining its more pronounced performance in continuous variable analysis. Furthermore, inconsistencies in confounder adjustment also contribute to the lack of statistical significance in OR analyses. Included studies varied substantially in their adjustment for key confounders such as eGFR, UACR, serum phosphate, inflammatory markers, and renin–angiotensin–aldosterone system (RAAS) inhibitors when calculating ORs. Evidence suggests that after comprehensive adjustment for renal function, the association between FGF23 and DKD may be attenuated, indicating that elevated FGF23 levels are partially driven by declining kidney function itself (18). In this context, the OR might reflect the weakened “independent effect” of FGF23, whereas the mean difference analysis better captures its overall elevation trend in the disease state. Lastly, heterogeneity in classification criteria cannot be ignored. Different studies lacked a uniform standard for defining “high FGF23 level” (e.g., based on quartiles, median, or empirical cut-off points). This arbitrariness in threshold setting significantly increases the instability of pooled OR analysis (32). In comparison, continuous variable analysis, which does not rely on artificial cut-offs, offers better comparability across studies.

Previous studies have reported significant associations between FGF23 and DKD and suggested its predictive value for DKD development (4, 5, 20, 27), which is consistent with our findings. Nevertheless, some studies have reached conclusions different from ours. Farías-Basulto et al. (21) reported that higher FGF23 levels were associated with a reduced risk of DKD. This finding should be interpreted with caution. Several factors may explain this seemingly contradictory association, particularly treatment-related influences. The widespread use of RAAS inhibitors, especially angiotensin receptor blockers (ARBs), may have altered the observed association. Increasing evidence suggests that RAAS blockade can influence the FGF23–Klotho–phosphate axis by restoring Klotho expression and modulating mineral metabolism, thereby potentially affecting circulating FGF23 levels (33). Moreover, a bidirectional interaction exists between FGF23 and RAAS: angiotensin II and aldosterone can stimulate FGF23 production, whereas elevated FGF23 may activate RAAS signaling through suppression of ACE2 (34–36). Given that ARBs are well established in improving renal outcomes (33), their dual regulatory effects on FGF23 levels and renal prognosis may introduce bias into observational associations. Under certain conditions, elevated FGF23 levels may represent an adaptive response aimed at temporarily alleviating metabolic disturbances rather than directly promoting renal injury. These factors highlight the context-dependent nature of the relationship between FGF23 and DKD and suggest that it may be influenced by treatment status, study design, and disease stage, thereby contributing to the heterogeneity observed in this meta-analysis. Ozdemir et al. reported a non-significant association between FGF23 levels and the occurrence of DKD (26). They reported a stronger association between Klotho levels and DKD. However, other studies have demonstrated a positive correlation between Klotho and FGF23 levels in patients with diabetes (21, 27). Moreover, the number of diabetic patients with eGFR <60 mL/min/1.73 m² in their study was relatively small, leading to classification into only two groups rather than five stages according to the Mogensen classification. This methodological limitation may have contributed to the divergent findings. Isakova et al. (18) suggested that the association between FGF23 and DKD is primarily driven by declining eGFR, with its predictive value markedly weakened after adjustment for renal function. However, they also reported that higher baseline FGF23 levels in patients with diabetes were independently linked to a higher risk of progressing to ESRD. The apparent inconsistency may stem from the study population, which consisted of non-randomized participants whose baseline serum samples were originally collected for trials examining cardiovascular outcomes under specific pharmacological interventions in T2DM. Thus, it is potentially unable to exclude whether a heavier comorbidity burden in the included patients masked underlying effects. Lee et al. (19) further noted that the predictive effect of FGF23 on DKD may be influenced by the inflammatory marker TNFR1, but FGF23 exerted an independent effect on all-cause mortality in T2 DM. This discrepancy may be attributable to the measurement of only C-terminal FGF23 rather than the intact molecule. In addition, Fayed et al. proposed the existence of an endothelial dysfunction factor that may precede FGF23 alterations during DKD progression (28). Given that FGF23 rises markedly with declining renal function (12, 31), and that their study primarily examined the relationship between FGF23 and flow-mediated dilation (FMD) in DKD, which is likely a major reason for the inconsistent conclusions.

FGF23 is predominantly secreted by osteocytes and osteoblasts (37, 38) and serves as a key regulator of calcium–phosphate homeostasis and vitamin D activation (39, 40). It participates in multiple pathological processes implicated in DKD, including insulin resistance, inflammation, and fibrosis (17, 41–43). Through complex signaling pathways, FGF23 exerts multifaceted effects on the kidney. Evidence indicates that FGF23 levels rise significantly as DKD progresses, enhancing phosphate excretion and thereby exposing residual renal tubules to potentially toxic phosphate concentrations—an effect related to compensatory responses during phosphate retention, as previously demonstrated (44, 45). In addition, eGFR levels may also contribute to the elevation of FGF23. This suggests that dynamic changes in FGF23 concentrations reflect the integrated regulation of renal metabolic processes. Clinical studies have also shown positive correlations between serum FGF23 levels and fibrosis markers in DKD patients, suggesting that FGF23 may contribute to renal fibrosis by modulating profibrotic mediators (46). Furthermore, FGF23 is closely linked to inflammatory states (47, 48), and chronic low-grade inflammation has been established as a central pathogenic mechanism of DKD (49). Dysregulation of FGF23 has been implicated in DKD pathogenesis (50), and inflammatory responses can upregulate FGF23 expression (47), indicating its involvement in inflammatory signaling pathways (41). Besides, excessive elevation of FGF23 has also been associated with increased cardiovascular risk (51). Extensive evidence demonstrates that FGF23 activates FGFR4 in cardiomyocytes, leading to left ventricular hypertrophy, myocardial fibrosis, metabolic remodeling, and electrophysiological abnormalities, which are key mechanisms underlying the high cardiovascular mortality observed in CKD (52–57). These effects may be partly mediated through indirect activation of the RAAS. Endothelial dysfunction may further link FGF23 and DKD, as FGF23 has been shown to reduce nitric oxide bioavailability by increasing asymmetric dimethylarginine and superoxide levels, thereby impairing endothelium-dependent vasodilation (58, 59).

Importantly, heterogeneity in baseline metabolic and biochemical characteristics among the included studies may have further influenced the observed association between FGF23 and DKD. As shown in **Table 1**, substantial differences existed in metabolic control indicators such as HbA1c and BMI in various studies. Poor glycemic control and obesity are closely linked to insulin resistance and chronic low-grade inflammation, both of which have been reported to stimulate FGF23 production (60). Therefore, elevated FGF23 levels in certain populations may partly reflect underlying metabolic disturbances rather than renal injury per se. In addition, mineral metabolism parameters—particularly serum phosphate—are key physiological regulators of FGF23 secretion (12, 45). Given the role of FGF23 as a compensatory phosphaturic hormone, even minor differences in phosphate levels across studies may result in significant variation in circulating FGF23 concentrations. This is particularly relevant in DKD, where phosphate retention occurs early and progressively worsens with declining renal function. Furthermore, inconsistent reporting of uric acid across studies represents another potential confounder. Uric acid has been associated with oxidative stress, endothelial dysfunction, and tubular injury (61), all of which may contribute to DKD progression and interact with FGF23-related pathways. The lack of uniform adjustment for these confounders across studies may partly explain the substantial heterogeneity observed and indicates that the relationship between FGF23 and DKD is influenced by complex interactions among metabolic and biochemical factors.

This systematic review and meta-analysis synthesized current evidence to clarify the association between FGF23 levels and DKD risk and provide insights into potential biological and clinical interest. Although elevated FGF23 levels were observed in patients with DKD, no significant association was found in dichotomous analyses, indicating that its role as an independent predictive biomarker remains uncertain. Therefore, FGF23 should not be considered a substitute for established renal markers such as eGFR and UACR. From a pathophysiological perspective, FGF23 reflects disturbances in mineral metabolism, inflammation, and bone–kidney endocrine signaling (11, 17, 50), aspects not fully captured by conventional renal function indicators. This suggests that changes in FGF23 levels may reflect certain metabolic alterations during the progression of DKD. However, given the extremely high and largely unexplained heterogeneity across studies, the clinical application of FGF23 is currently not supported by sufficient evidence and should be interpreted with caution, and its incremental value over established biomarkers remains uncertain.

Carried out in strict compliance with PRISMA guidelines, this comprehensive systematic review and meta-analysis is nevertheless subject to several limitations. First and foremost, the continuous variable analysis revealed extremely high and largely unexplained heterogeneity. Although subgroup analyses were performed, the specific sources of heterogeneity could not be clearly identified, and the relatively small number of included studies may have further exacerbated this limitation. This represents a major methodological concern and substantially reduces the reliability of the pooled effect estimates. Furthermore, methodological differences among the included studies may also represent a source of heterogeneity. These include variations in FGF23 measurement methods (e.g., assay manufacturers, detection ranges, sampling timing, and sample storage conditions) as well as differences in the target fragments measured. Specifically, some studies assessed C-terminal FGF23, which detects both intact hormone and its cleavage fragments, whereas others measured intact FGF23, the biologically active form. Given that FGF23 cleavage may be altered under conditions such as inflammation or iron deficiency, these methodological inconsistencies may affect the comparability of measured FGF23 levels across studies and contribute to the observed heterogeneity. It should also be emphasized that pooled comparisons across different assay platforms should be interpreted with caution. Importantly, the use of different FGF23 assay platforms (e.g., C-terminal versus intact assays) across studies represents a critical methodological limitation. These assays may capture biologically distinct components of FGF23, thereby limiting true biological comparability between studies. Consequently, the pooled estimates may reflect not only underlying biological variation but also assay-related differences. This issue may not only reduce comparability but also introduce systematic bias in pooled effect estimates, potentially affecting both the magnitude and direction of the observed associations. This limitation may have contributed to the substantial heterogeneity observed and reduces the overall reliability of the findings. Therefore, this issue should be regarded as a limitation of the evidence base rather than merely a technical consideration. Second, baseline information varied across studies, and key variables such as DKD stage, serum uric acid, and eGFR were not consistently reported, precluding additional subgroup analyses. When interpreting the association between FGF23 and DKD, confounding factors must be carefully considered. Circulating FGF23 levels are influenced by multiple physiological and pathological factors, including serum phosphate, vitamin D levels, parathyroid hormone (PTH), inflammatory activity, and the use of RAAS inhibitors. These variables are closely related to mineral metabolism and renal function and may act as important confounders. In this study, adjustment for these factors was limited due to the lack of individual participant data. Moreover, adjustment strategies varied considerably across studies, with some controlling for renal function (e.g., eGFR, UACR) and metabolic parameters, while others reported unadjusted estimates. This inconsistency may have contributed to the observed heterogeneity and partially explains the attenuation of associations in pooled OR analyses. Third, substantial differences existed in participant age, sample size, ethnicity, geographic region, recruitment setting (community vs hospital), demographic data collection methods, DKD diagnostic criteria, exclusion criteria, duration of diabetes, presence of healthy controls, comorbidity profiles, and concomitant medication use. Differences in medication regimens across the included studies may also exist, and the pooled effect estimates reflect average associations under varying treatment conditions. This heterogeneity may have diluted the underlying biological association between FGF23 and DKD to some extent. Fourth, methods of biological sample collection varied, such as whether blood and urine samples were collected fasting, processed on the same day, and storage conditions. Fifth, because individual-level data were unavailable, covariate adjustment could not be performed, and confounding effects were not fully controlled. The OR analysis was based on only four studies, and thus current evidence is insufficient to robustly support FGF23 as a dichotomous predictor of DKD risk. In addition, inconsistent definitions of “high” and “low” FGF23 levels across studies further limited its predictive utility. Future research should standardize FGF23 assays to establish clinically meaningful cutoff values. Sixth, certain studies adopted two-stage designs involving cross-sectional analyses followed by prospective observational cohorts, and the inclusion of combined predictive models may also have affected outcomes. Besides, although Egger’s test did not indicate significant publication bias, the relatively small number of included studies may have reduced the statistical power of this test. Seventh, the statistical tools used for data analysis differed across studies, and averaging repeated measurements could also be a source of variation. In several studies, numerical data were not explicitly reported and were extracted from graphical representations using digitization tools. Although this process was independently conducted and cross-validated by two investigators, estimation from images may introduce measurement error and represent a potential source of heterogeneity. Nevertheless, sensitivity analyses confirmed that no single study substantially influenced the overall results, supporting the robustness of the main finding that elevated FGF23 levels are associated with DKD. Eighth, in some studies numerical data were not explicitly reported but presented graphically. Using digitizer tools for extraction might introduce errors. Ninth, given the limited number of included studies, meta-regression was not performed due to insufficient statistical power and the increased risk of false-positive findings. Additionally, the observational nature of all included studies (most of which were case–control designs) precludes causal inference. Therefore, the findings should be interpreted as associative rather than causal. Reverse causation remains plausible, as declining renal function during DKD progression may lead to phosphate retention and compensatory increases in FGF23 secretion. In this context, elevated FGF23 levels may reflect impaired kidney function rather than serving as a primary driver of the disease. Finally, whether studies were an independent trial or nested within other trials is also an important consideration. Besides, although subgroup analyses by DKD stage were performed, statistically significant associations were observed only in stages 2 and 4, suggesting that the strength of association may vary across disease stages. Current evidence is insufficient to fully support the utility of FGF23 for early DKD detection. Its predictive signal in stage 2 may overlap with the stage at which traditional biomarkers already become abnormal. Notably, subgroup analyses did not consistently demonstrate a significant association in the earliest stages of DKD. This inconsistency suggests that the utility of FGF23 for early detection remains uncertain. Therefore, these findings should be interpreted with caution, and FGF23 should not currently be considered a reliable biomarker for early-stage DKD identification. Additionally, the number of studies included in certain subgroups of this analysis was small. Analyses based on limited data typically result in wide confidence intervals and low statistical power. Therefore, the findings from these specific subgroup analyses should be considered exploratory and hypothesis-generating rather than conclusive.

Although FGF23 has emerged as a promising biomarker for DKD development and progression, its path to clinical translation remains constrained by the current evidence. To strengthen this foundation, future research should prioritize large-scale, multicenter prospective cohort studies (32, 62) to systematically evaluate the causal relationship between FGF23 and DKD outcomes across diverse populations defined by ethnicity, geography, and diabetes subtype, thereby minimizing the impact of sample bias on conclusion stability. Standardization remains a major bottleneck. Harmonizing FGF23 measurement methodologies, confounder adjustment strategies, and clinical endpoint definitions is essential to improve comparability and reproducibility across studies (29, 30). FGF23 may reflect underlying metabolic disturbances and renal pathological changes in DKD; however, its role in early detection or risk stratification remains uncertain and should be considered hypothesis-generating rather than clinically established.

## 5 Conclusion

In conclusion, our findings indicate that FGF23 levels are associated with the progression of DKD, suggesting that it may reflect metabolic disturbances associated with DKD. However, its role as an independent predictor of DKD risk remains unclear, and further well-designed prospective studies are needed to determine whether FGF23 provides incremental value beyond established renal biomarkers. In light of the substantial and largely unexplained heterogeneity, the findings of this study should be regarded as exploratory evidence and are not sufficient to support clinical application.

## Data Availability

The original contributions presented in the study are included in the article/**Supplementary Material**. Further inquiries can be directed to the corresponding author.

## Supplementary material

Supplementary File S1 Search strategy.
