# Supplementary material for: Fibroblast growth factor 23 levels and the risk of diabetic kidney disease: a systematic review and meta-analysis
Source: Front Endocrinol (Lausanne). 2026 May 19;17:1799186. doi: 10.3389/fendo.2026.1799186 (PMC13226003; doi:10.3389/fendo.2026.1799186)
Supplement: Supplementary File S1 — Search strategy. [file Table1.docx]

**File S1** Search strategy

| Pubmed | |
| --- | --- |
| # | Query |
| 1 | Diabetic Nephropathies[MeSH Terms] |
| 2 | "diabetes nephropathy"[Title/Abstract] OR "Diabetic  Glomerulosclerosis"[Title/Abstract] OR "diabetic kidney  disease*"[Title/Abstract] OR "diabetic nephropath*"[Title/Abstract] OR  "diabetic renal disease"[Title/Abstract] OR "Intracapillary  Glomerulosclerosis"[Title/Abstract] OR "Kimmelstiel Wilson  Disease"[Title/Abstract] OR "Kimmelstiel Wilson Syndrome"[Title/Abstract]  OR "Nodular Glomerulosclerosis"[Title/Abstract] |
| 3 | #1 OR #2 |
| 4 | Fibroblast Growth Factor-23[MeSH Terms] |
| 5 | "FGF 23"[Title/Abstract] OR "FGF23"[Title/Abstract] OR "fibroblast growth  factor 23"[Title/Abstract] OR "phosphatonin"[Title/Abstract] OR "tumor  derived hypophosphatemia factor"[Title/Abstract] OR "tumor derived  hypophosphatemia inducing factor"[Title/Abstract] OR "tumour derived  hypophosphataemia factor"[Title/Abstract] OR "tumour derived  hypophosphataemia inducing factor"[Title/Abstract] |
| 6 | #4 OR #5 |
| 7 | #3 AND #6 |

| Embase | |
| --- | --- |
| # | Query |
| 1 | 'diabetic nephropathy'/exp |
| 2 | 'diabetes nephropathy':ti,ab,kw OR 'diabetic glomerulosclerosis':ti,ab,kw OR  'diabetic kidney disease*':ti,ab,kw OR 'diabetic nephropath*':ti,ab,kw OR  'diabetic renal disease':ti,ab,kw OR 'intracapillary  glomerulosclerosis':ti,ab,kw OR 'kimmelstiel wilson disease':ti,ab,kw OR  'kimmelstiel wilson syndrome':ti,ab,kw OR 'nodular  glomerulosclerosis':ti,ab,kw |
| 3 | #1 OR #2 |
| 4 | 'fibroblast growth factor 23'/exp |
| 5 | 'fgf 23':ti,ab,kw OR 'fgf23':ti,ab,kw OR 'fibroblast growth factor 23':ti,ab,kw  OR 'phosphatonin':ti,ab,kw OR 'tumor derived hypophosphatemia  factor':ti,ab,kw OR 'tumor derived hypophosphatemia inducing  factor':ti,ab,kw OR 'tumour derived hypophosphataemia factor':ti,ab,kw OR  'tumour derived hypophosphataemia inducing factor':ti,ab,kw |
| 6 | #4 OR #5 |
| 7 | #3 AND #6 |

| Cochrane Library | |
| --- | --- |
| # | Query |
| 1 | MeSH descriptor: [Diabetic Nephropathies] explode all trees |
| 2 | ('diabetes nephropathy' OR 'Diabetic Glomerulosclerosis' OR 'diabetic  kidney disease*' OR 'diabetic nephropath*' OR 'diabetic renal disease' OR  'Intracapillary Glomerulosclerosis' OR 'Kimmelstiel Wilson Disease' OR  'Kimmelstiel Wilson Syndrome' OR 'Nodular Glomerulosclerosis'):ti,ab,kw |
| 3 | #1 OR #2 |
| 4 | MeSH descriptor: [Fibroblast Growth Factor-23] explode all trees |
| 5 | ('FGF 23' OR 'FGF23' OR 'fibroblast growth factor 23' OR 'phosphatonin' OR 'tumor derived hypophosphatemia factor' OR 'tumor derived  hypophosphatemia inducing factor' OR 'tumour derived hypophosphataemia  factor' OR 'tumour derived hypophosphataemia inducing factor'):ti,ab,kw |
| 6 | #4 OR #5 |
| 7 | #3 AND #6 |

| Web of Science | |
| --- | --- |
| # | Query |
| 1 | "TS=((diabetes nephropathy) OR (Diabetic Glomerulosclerosis) OR (diabetic  kidney disease*) OR (diabetic nephropath*) OR (diabetic renal disease) OR  (Intracapillary Glomerulosclerosis) OR (Kimmelstiel Wilson Disease) OR  (Kimmelstiel Wilson Syndrome) OR (Nodular Glomerulosclerosis)) " |
| 2 | "TS=((FGF 23) OR (FGF23) OR (fibroblast growth factor 23) OR  (phosphatonin) OR (tumor derived hypophosphatemia factor) OR (tumor  derived hypophosphatemia inducing factor) OR (tumour derived  hypophosphataemia factor) OR (tumour derived hypophosphataemia  inducing factor))" |
| 3 | "#1 AND #2 " |
